# Development of a schwarzite-based moving bed 3D printed water treatment system for nanoplastic remediation†

**DOI:** 10.1039/d1ra03097c

**Published:** 2021-06-01

**Authors:** Bramha Gupta, Rushikesh S. Ambekar, Raphael M. Tromer, Partha Sarathi Ghosal, Rupal Sinha, Abhradeep Majumder, Partha Kumbhakar, P. M. Ajayan, Douglas S. Galvao, Ashok Kumar Gupta, Chandra Sekhar Tiwary

**Affiliations:** School of Water Resources, Indian Institute of Technology Kharagpur Kharagpur 721302 India; Metallurgical and Materials Engineering, Indian Institute of Technology Kharagpur Kharagpur 721302 India chandra.tiwary@metal.iitkgp.ac.in; Applied Physics Department, State University of Campinas – UNICAMP 13083-859-Campinas SP Brazil galvao@ifi.unicamp.br; School of Environmental Science and Engineering, Indian Institute of Technology Kharagpur Kharagpur 721302 India; Department of Materials Science and Nanoengineering, Smalley-Curl Institute, Rice University Houston Texas 77005 USA; Environmental Engineering Division, Department of Civil Engineering, Indian Institute of Technology Kharagpur Kharagpur 721302 India agupta@civil.iitkgp.ac.in

## Abstract

The impact of micro and nanoplastic debris on our aquatic ecosystem is among the most prominent environmental challenges we face today. In addition, nanoplastics create significant concern for environmentalists because of their toxicity and difficulty in separation and removal. Here we report the development of a 3D printed moving bed water filter (M-3DPWF), which can perform as an efficient nanoplastic scavenger. The enhanced separation of the nanoplastics happens due to the creation of a charged filter material that traps the more surface charged nanoparticles selectively. Synthetic contaminated water from polycarbonate waste has been tested with the filter, and enhanced nanoplastic removal has been achieved. The proposed filtration mechanism of surface-charge based water cleaning is further validated using density function theory (semi-empirical) based simulation. The filter has also shown good structural and mechanical stability in both static and dynamic water conditions. The field suitability of the novel treatment system has also been confirmed using water from various sources, such as sea, river, and pond. Our results suggest that the newly developed water filter can be used for the removal of floating nanoparticles in water as a robust advanced treatment system.

## Abstract 2

The impact of micro and nanoplastic debris on our aquatic ecosystem is among the most prominent environmental challenges we face today.

## Graphical abstract

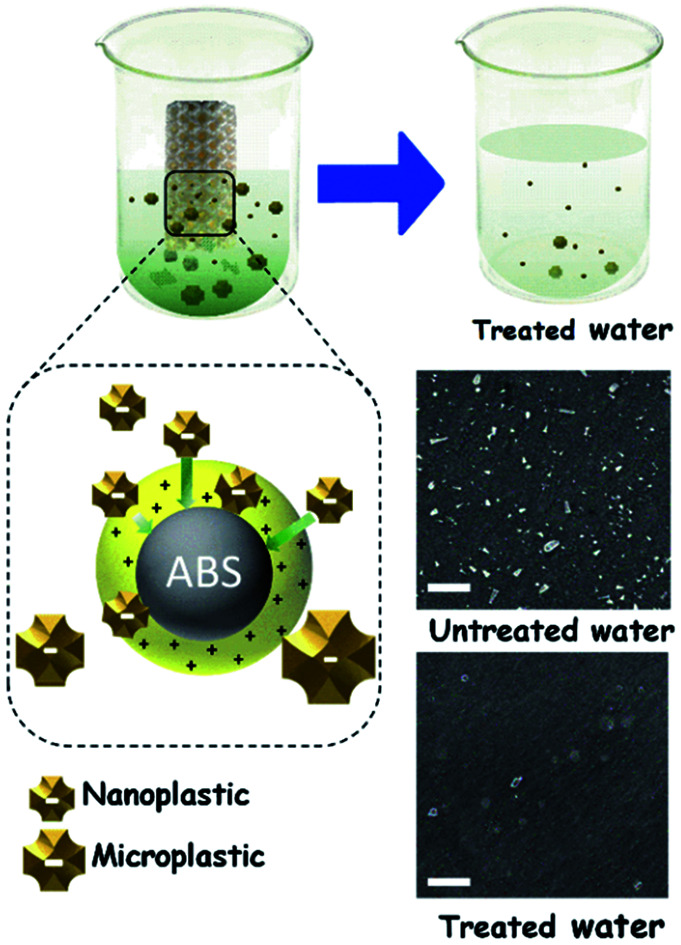

## 1 Introduction

The extensive use of plastics and their subsequent unplanned disposal have made them one of the most notable water pollutants in the last few decades. In 2015, about 6.3 billion tonnes of plastic waste production was reported globally, out of which only 9% was recycled, 12% was incinerated, and 79% was dumped in landfills or water bodies.^1^ The size of the disposed plastics is reduced during fragmentation, aggregation, deposition, and transportation in water bodies.^2–6^ As plastics are ubiquitous and long-lasting, they become accumulated rather than decomposed.^1,7,8^ Based on the size of the plastics, they can be categorized as microplastics (more than 100 nm up to 500 μm) and nanoplastics (<100 nm).^9–12^ The larger plastics eventually get settled, but the micro and nanosized plastics can float on the surface of water bodies. At present, the impact of micro and nanoplastics debris on the aquatic ecosystem is considered one of the most increasing problems. Nanoplastics have become a global concern for environmentalists because of their toxicity towards the biosphere. This persistent organic pollutant can further absorb other contaminants and promotes “bio-accumulation” and “bio-magnification”.^13–15^ Nanoplastics can inhibit the growth of cells, reduce cellular chlorophyll concentration, lower reproduction rate, cause severe development defects, and affect metabolism among various microorganism.^16^ These plastics can be consumed by human beings through direct (drinking water) and indirect (propagation in food-chain by consuming aquatic organisms like fishes and prawns) pathways.^1^

Advanced physical treatment processes, such as dissolved air floatation, rapid sand filter, disc filters, and biological processes, have been employed to remove microplastics from the wastewater.^17–21^ The average removal efficiency using disc filters was found to be around 40%, while dissolved air floatation and rapid sand filtration techniques accounted for removal of around 48% and 90%, respectively.^17^ Filtration is considered an effective technique for the removal of microplastics due to its trapping and adsorption ability. On the other hand, for separation of nanoplastics using the conventional filters have several limitations, such as low filtration efficiency, poor mechanical resistance, and pore blockage.^22^ The filtration efficiency is improved with the help of increasing surface area of the porous architecture.^23^ The porous filters are fabricated by gas foaming, solvent casting/particulate leaching, freeze-drying, and 3D printing^24–29^ techniques. Among these, 3D printing is the competitive technique due to its numerous advantages, such as its capability to fabricate complex structures, agility to print a wide spectrum of compatible materials, sustainability, and scalability.^30–36^ Furthermore, a schwarzite-based 3D structured porous filter was used due to its large surface area, positive and negative curving topologies with tunable spongy size and shape, and intriguing properties. These crystalline constructions can have a large number of porous unit cells, rigid foam-like materials with tunable mechanical and electronic properties.^27,37^

The separation of nanosized particles is more difficult compared to the higher-sized particles in the conventional filtration process, which creates significant difficulties in nanoplastics removal from an aqueous solution. We determined that the selectivity towards nanosized particles for a charged filter media can be developed from the fact that the surface charge of the particles increases with the decreasing particle size, which can be due to the surface area effect.^38,39^ Thus, the development of a novel nanoplastics removal system can be exploited from this innovative separation concept.

**Fig. 1:**
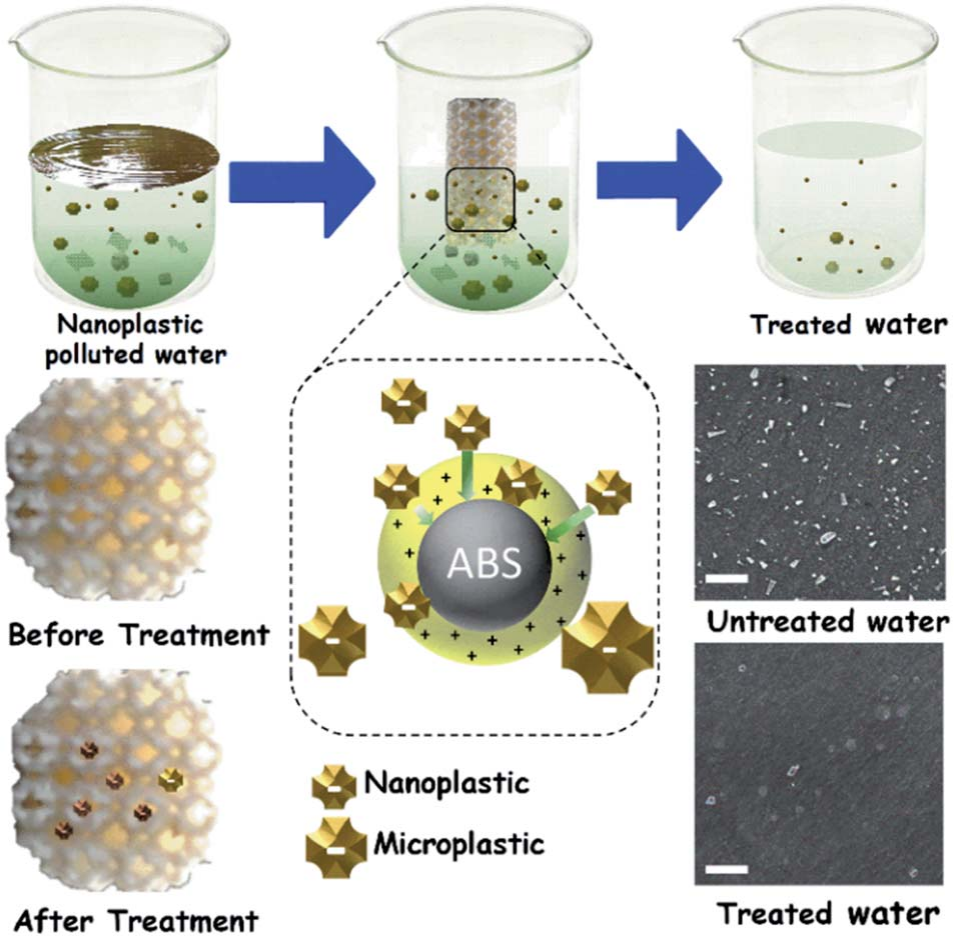
Scheme of the M-3DPWF preparation, its use for nanoplastics filtration, physicochemical assessment of the filtration performance, and filtration mechanism. Scale bar 500 nm.

**Fig. 2:**
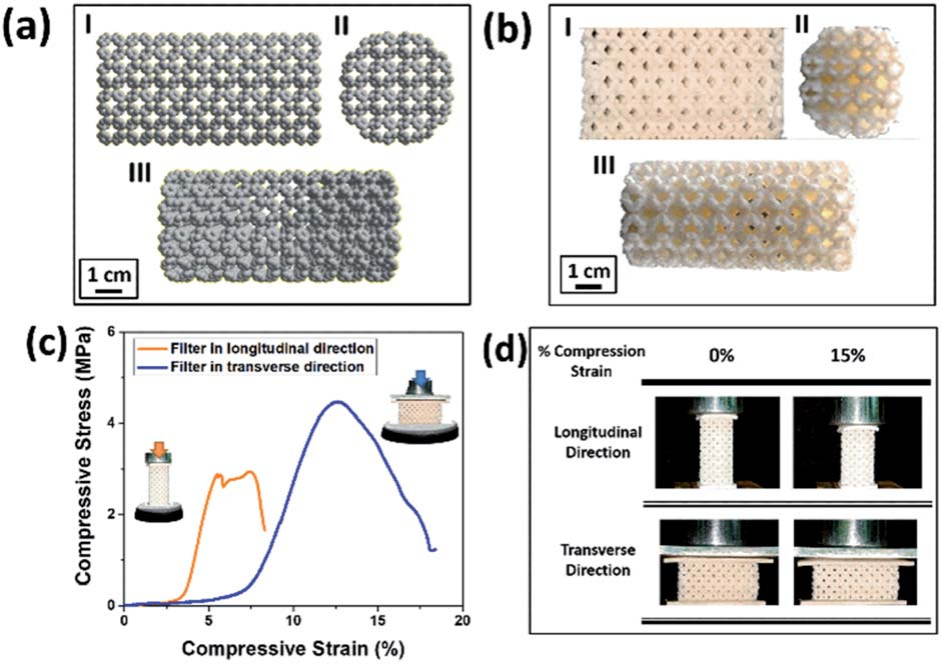
(a and b) Atomistic model from molecular dynamics simulations and the corresponding ABS 3D printed micro-model one (I: front view; II: top view; III: isometric view). (c) Curves for the uniaxial compressive tests along longitudinal and transverse filter directions. (d) Representative snapshots of the 3D printed model at specific deformation percentages along longitudinal and transverse directions.

In this study, we have fabricated a novel 3D printed moving bed water filter (M-3DPWF) targeting the filtration of nanoplastics (as shown in Fig. 1). The structural ability of the M-3DPWF was tested by measuring its compressive stress, yield stress, and toughness (as depicted in Fig. 2). The M-3DPWF was employed to treat polycarbonate contaminated synthetic water. Microscopy and spectroscopy of the M-3DPWF treated water were also carried out to confirm the nanoplastics removal. The field suitability of the M-3DPWF was further tested using source water from the sea, river, and pond. The proposed mechanism of surface charge-based water cleaning is further supported using DFT-based simulation. The proposed technology can be an advanced and feasible solution for the concerns of nanoplastics contamination in water bodies worldwide.

## 2 Experimental

### 2.1 Experimental details

The chemicals required for the preparation of M-3DPWF is commercial grade acrylonitrile butadiene styrene (ABS) (ABS material was selected due to its higher molar attraction constant ∼2384 J^1/2^ cm^3/2^ mol^−1^)^40^ supplied by WOL3D from Mumbai, India, having a diameter of 1.75 mm with a tolerance of ±0.1 mm. The ABS filament has a density of 1.31 g cm^3^ and melts at 210 °C. Tensile and compressive strength of solid ABS ranges from 32–45 MPa and 65–90 MPa respectively. ABS is also popular for its good impact resistance and heat resistance capabilities.^41^ Polycarbonate sheet (3 mm thickness, 150 × 150 mm size) was purchased from Sigma Aldrich for the preparation of nanosized polycarbonate particles. All the reagents were prepared using deionized water. In Fig. 1, we present the scheme of M-3DPWF fabrication using ABS, the presence of nanoplastics in water (along with SEM image), its filtration using M-3DPWF, the physicochemical characterization of the treated water showing the removal of nanoplastics, and the filtration mechanism. Schwarzites are mathematically, 3D porous solids with periodic minimal surfaces having negative Gaussian curvatures. The optimized primitive schwarzite structures (which have trigonal voids/holes connected on their edges) have been selected due to their higher compressive strength than other schwarzite structures (*i.e.*, gyroid).^27^ These structures were obtained from molecular dynamics simulations [front, top, and the isometric view is shown in Fig. 2a(I–III)] and converted into .stl format, after which the .stl file converted to g-code format using FlashPrint 4.1.0 slicer. The FlashForge Adventure 3 Printer (Fused Deposition Modeling) were utilized to fabricate porous filters. The ABS filament utilizes to print the filter was melted in the extruder at 210 °C and extruded through a nozzle onto the heated bed. The temperature of the heated bed was maintained at 60 °C throughout the printing process. The novel structure had a single-layer height of 0.18 mm along the *Z*-direction. The structure exhibits notable porosity, therefore fill density was kept at 100%. The 3D printer is popular for the high speed of the printing. The travel speed was maintained at 75 mm s^−1^, and print speed was kept at 55 mm s^−1^. The rapid solidification of molten extruded mass was controlled by a cooling fan. The printed filter (front, top, and the isometric view) is shown in Fig. 2b(I–III). The structural analysis (compressive stress, yield stress, and toughness) of the novel M-3DPWF was done using a universal testing machine (A.G. 5000G, Shimadzu) with a 1 mm min^−1^ constant displacement rate. Water absorption tests were carried out as per the ASTM D 570 standard (in stationary and dynamic conditions). Before testing, the specimens were kept in an oven at 23 °C for 24 h. They were kept under the quiescent condition for stationary testing and continuous stirring at 300 rpm for 24 h for dynamic testing for water absorption. Among the several plastic materials available in the world, polycarbonate is one of the widely used plastics having a persistent and toxic nature. In this study, polycarbonate was used as a target pollutant and was cut into small size rectangular plates (1 cm × 1 cm) and then reduced to particles (micro and nano) by grinding it in the mixer (500 W, Havells, India). The obtained grounded particles (micro and nano) after grinding were homogeneously mixed in deionized water by stirring at 300 rpm. The M-3DPWF block was immersed into a glass beaker containing 200 ml of plastics polluted water and kept under the stationary condition for 48 h to complete the filtration activity. The sample was collected from the top surface for further spectroscopic and microscopic analysis. Similarly, the filtration experiment was also performed on source water (sea, river, and pond samples).

The presence of nanoplastics in the water samples was assessed by measuring its particle size distribution in water before and after treatment conditions. Particle concentration in the treated water was measured using a Zetasizer, which corresponds to the removal of nanoplastics. Spectroscopic and microscopic analyses of the contaminated water at both the initial and final stages were also performed to get the physicochemical attributes of the nanoplastics. The Fourier transform infrared (FTIR) analysis of the source, raw, and treated water was carried out using FTIR spectroscopy (Nicolet-6700, Thermo Fisher, USA), having wavenumbers ranging from 250 to 4250 cm^−1^. The surface morphology was assessed by field-emission scanning electron microscopy (FE-SEM) analysis (ZEISS-MERLIN, GEMINI-2, Germany). The sample preparation procedures for FTIR and FE-SEM analysis have been given in ESI (Section S1†). High-resolution transmission electron microscopy (HR-TEM) was carried out to obtain the in-depth surface morphology and size distribution at the nanoscopic level (JEM-2100F, Make-JEOL, Japan). Thermogravimetric analysis (TGA) was performed using a thermogravimetric/differential thermal analyzer (Perkin Elmer Pyris Diamond TG/DTA, USA) by introducing nitrogen gas and increasing temperature at 10 °C min^−1^. Zeta potential and average particle size of the treated water were measured using a Zetasizer Nano ZS90 analyzer (Malvern, UK).

### 2.2 Simulation details

In order to investigate the electrostatic interaction between the polymer chains (ABS and polycarbonate) discussed in the Experimental section of this manuscript, we considered two structures of ABS (35 atoms) and polycarbonate (PC) (35 atoms) molecules (see Fig. 5I). For each configuration of the ABS (PC) molecule located at the origin, we calculated the configuration interaction energy in terms of separation between ABS (PC) and PC (ABS) molecule for different configurations. This configuration energy is given by:

1 *E* = *E*_AB_ − *E*_A_ − *E*_B_

where *E*_AB_ is the total energy obtained when the ABS (PC) interacts with the PC (ABS), *E*_A_ and *E*_B_ are the total energy obtained for the ABS (PC) isolated configurations. We used the semi-empirical MOPAC2016 software to carry out the geometrical optimizations and obtain each component in eqn (1). Semi-empirical methods, in general, produce the interaction energy or heats of formation with high accuracy for large systems, in particular, the ones composed of carbon, hydrogen, oxygen, and nitrogen atoms. The most recent parameterization method available in MOPAC2016 is PM7.^42^ Until recently, the weak interaction as hydrogen bond and van der Waals interactions were not well described. But the last modifications in the PM6 and PM7 parameterization have been producing results with very high accuracy,^43,44^ including large systems (thousands of atoms).^45^ PM7 was applied to obtain the optimized geometries, configuration energy, and charges (electrostatic maps). We tested for other comparable parameterizations available in the MOPAC2016, such as PM6 and PM6-DH2, but the results were nearly the same. Therefore, we expect that PM7 will accurately describe the charge redistribution due to the electrostatic interactions between ABS and PC.

## 3 Results and discussions

In Fig. 2a, we present the optimized atomistic schwarzite model obtained from molecular dynamics simulations. It was used to generate the macroscale model to be 3D printed into a filter form named as moving bed 3D printed water filter (M-3DPWF) (Fig. 2b). There are two types of spherical porosity present in the filter with a diameter of 0.12 and 0.30 cm, we have also calculated the theoretical surface area of the filter *via* Blender 2.82 software which is 1000.69 cm^2^. The physical properties of this highly porous architecture have been evaluated. The compression tests showed that M-3DPWF has similar yield strength values for the transverse and longitudinal directions were 4 MPa and 2.5 MPa, respectively (Fig. 2c). The M-3DPWF absorbed energy along the longitudinal and transverse directions were found to be 0.0365 J m^−3^ and 0.1045 J m^−3^, respectively. The observed anisotropic M-3DPWF behavior is due to its structural topology, such that the faster load transfer occurs in the transverse direction as compared to the longitudinal one. This is a common behavior of some schwarzite families.^27,37^ During the compression tests, the real-time images at different strain intervals (% strain) show that along the longitudinal direction, pore deformation starts from 5% onwards, while along the transverse direction, it starts only from 15% onwards, as shown in Fig. 2d. We present the M-3DPWF water absorption capability under stationary and dynamical regimes. In the stationary regime, M-3DPWF absorbed 11.43% water, while in the dynamical one (continuous stirring for 24 h), it absorbed 13.42% water. These results indicated that the water filter is highly stable in the stationary as well as in dynamical conditions. Recently, there were several reports on the fabrication of porous architecture of polymers and ceramic hybrids synthesized using conventional processes^27,46–49^ for filters. In all these filters, the increase in porosity reduces strength and increases water absorption due to poor control over pore size and topology (Table S1 in the ESI†). The combination of strength and water absorption in the current 3D printed filter is superior as compared to other pre-existing filters.

**Fig. 3:**
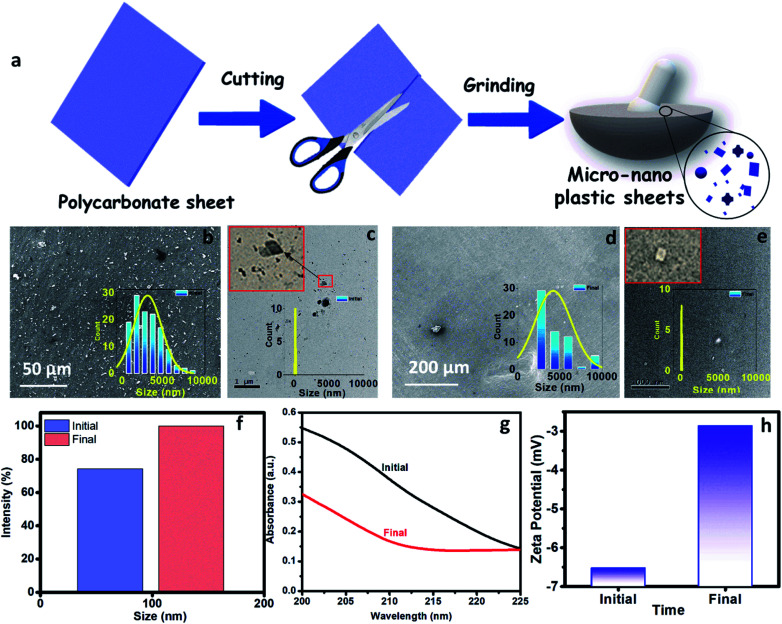
(a) Preparation of nanosized plastics by the cutting and grinding of the polycarbonate sheet. (b and d) Field-emission scanning electron microscopy images of the initial and treated water. Inset shows the Histogram of micrograph images showing the size range of the microplastics. (c and e) High-resolution transmission electron microscopy images of the initial and treated water. Inset shows the Histogram of TEM images showing the size range of the nanoplastics. (f) Zeta sizer analysis of the initial and treated water to show the average size of the available particles. (g) UV absorbance of the initial and treated water. (h) Zeta potential of the initial and treated water to show its particle charges in the solution.

The M-3DPWF was used to treat nanosized polycarbonate polluted water. The spectroscopy and microscopy analyses of the treated water were carried out to obtain the filtration performance of the 3D printed water filter. In Fig. 3a, the schematic shows the preparation process of polycarbonate nanoparticles. The FTIR spectra (Fig. S1a, d, and e, ESI†) of the initial and treated water show the presence of the chemical bonds of the polycarbonate plastics in the solution, as discussed in the ESI (Section S2).† There is no peak shift in the FTIR spectra of the initial and final solution, which confirms that there is no change in the chemical behavior of polycarbonate during the removal process. In Fig. 3b and d we present the FE-SEM images of the initial and treated water. The low magnification SEM image of the polluted water shows a large number of flake-like polycarbonate particles (Fig. 3b). The microscopic size analysis of polluted water [as shown in histogram (inset of Fig. 3b)] reveals the particles were in the size range of 1000–9000 nm, with the majority of the particles being below 3000 nm. The HRTEM analysis of polluted water (Fig. 3c) shows the presence of 25–160 nm-sized polycarbonate particles with most particles being below 70 nm [as shown in histogram (inset of Fig. 3c)]. The FE-SEM of treated water (Fig. 3d) shows a drastic reduction in the number of observed particles (1000–9000 nm). The few particles observed in the micrograph are of 9000–10 000 nm size range [as shown in the inset of Fig. 3d)]. The HRTEM of treated water has also shown a drastic decrease in the number of observed particles (25–160 nm) (Fig. 3e). Even in the histogram of treated water (inset of Fig. 3e), it can be seen that particles up to size 60 nm are absent. These results confirm that the M-3DPWF is efficient to remove nano (majorly) and micro-size polycarbonate particles from the water. The Zetasizer analysis (Fig. 3f) shows that the frequency of particle size distribution predominantly varies in the range of 70–105 nm, wherein around 90 nm sized particles occur most frequently in the polluted water. On the other hand, the frequency distribution of particles present in the treated water was shifted to a higher range (90–125 nm) with the maximum occurring particles of size ∼110 nm. The bar diagram of treated water exhibited the drastic reduction of smaller size polycarbonate particles, which is ascribed to the filtration ability of the M-3DPWF in the removal of the nanosized polycarbonate plastics (Fig. 3f). Additionally, from Fig. S2,† it can be seen that in raw water the maximum number of particles (about 50%) is of size about 190 nm (Fig. S2a†), while in treated water the maximum number of particles (about 40%) are of size more than 400 nm (Fig. S2b†), which confirms the separation of polycarbonate nanoparticles using the M-3DPWF. Furthermore, from Fig. S3,† it can be observed that the distribution of the particles in the different samples of polycarbonate for initial and treated water are varying. For example, for treated water, the sample was collected from the top, middle, and bottom locations, which is showing the average particle size of, ∼30 nm, ∼51 nm, and ∼106 nm, respectively. Whereas, for three initial samples, it was ∼87 nm, ∼91 nm, and ∼165 nm, respectively.

The removal of polycarbonate particles from the polluted water was further validated using UV absorbance data, as shown in Fig. 3g. The absorbance of the treated water is lesser than the nanoplastics polluted water. The presence of more nanoparticles in the polluted water will block the path of UV light, which is responsible for its higher absorbance. The reduced absorbance of the treated water is attributed to the filtration ability of the M-3DPWF for the removal of polycarbonate particles (nano and micro). To test the maximum filtration ability of the 3D water filter, filtration of polycarbonate polluted water was further performed above 48 h. From Fig. S4,† it is observed that the absorbance of the treated water did not significantly change at 60 h, hence it can be concluded that the 3D water filter is showing the maximum filtration ability at 48 h. From the zeta potential plot (Fig. 3h), it can be observed that the zeta potential is lower for the treated solution (∼−3.0 mV) than the initial solution (∼−6.5 mV). The decrease in the zeta potential can be attributed to the removal of charged polycarbonate (small-sized) particles through filtration.

**Fig. 4:**
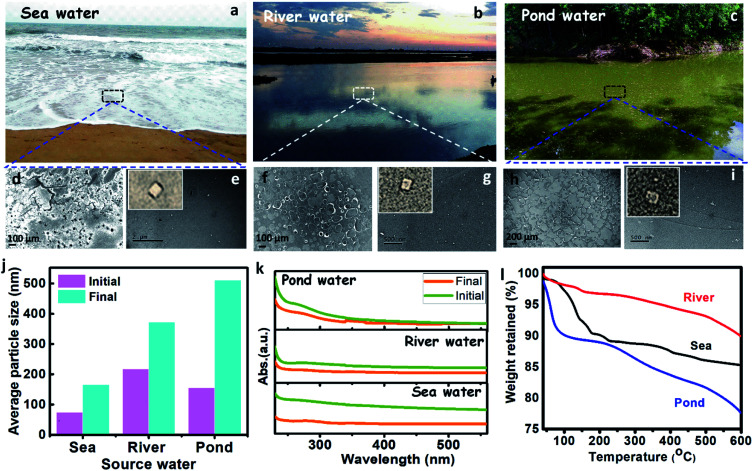
(a–c) Digital photographs of the source water such as sea, river, and pond water. (d, f, and h) Field-emission scanning electron microscopy images of the sea, river, and pond water. (e, g, and i) High-resolution electron microscopy images of the sea, river, and pond water. (j) Zetasizer analysis of the sea, river, and pond water to show the average size of the available particles at the initial and final stage. (k) UV-vis absorbance of the river, and pond water at the initial and final stage. (l) Thermogravimetric analysis of the sea, river, pond water.

The presence of plastics (micro and nano) in the different source water, such as sea, river, and pond were assessed by performing spectroscopic and microscopic analyses. The source water was collected from the Bay of Bengal (Digha, East Midnapore, West Bengal, 21°37′35.8212′′N 87°30′26.7516′′E), Hooghly River (Konnagar, Hooghly, West Bengal, 22°42′18.3′′N 88°20′40.1′′E), and Black Lake (IIT Kharagpur, Kharagpur, West Midnapore, West Bengal, 22°19′6.58′′N 87°18′21.52′′E), respectively. The collected water was preserved, and subsequently, its physicochemical analysis was carried out. The images of source water (sea, river, and pond) are shown in Fig. 4a–c. The FTIR spectra of the source water (Fig. S1b, c, and f, ESI†) have shown the presence of polyethylene, polypropylene, polyvinyl chloride, nylon-6, polystyrene, and polycarbonate, as discussed in the ESI (Section S1).†

The FE-SEM analysis (Fig. 4d, f, and h), revealed that sea, river, and pond water samples contain flakes and granular-like particles. The presence of dark patches in the FE-SEM images may belong to organic solids in water. The histogram of the micrograph (Fig. S5a–c†) displays the presence of maximum particles up to the size of 10 000, 40 000, and 100 000 nm for sea, river, and pond water, respectively. The HRTEM analysis of source water (sea, river, and pond) has shown the presence of plastic particles (bright particles) along with other organic solids (Fig. 4e, g, and i). Further, the histogram of HRTEM analysis (Fig. S5d–f†) shows that most particles are found up to size 70, 30, and 8 nm for sea, river, and pond water, respectively. These results confirm that the seawater contains nanoparticles in a larger size range (∼70 nm) and microparticles in a smaller size range (∼10 000 nm) than those in river and pond water. The zeta size analysis shows that the average size of the particles in the sea, river, and pond water is 73.82, 217.1, and 155.6 nm, respectively, as shown in Fig. 4j, which confirms that the nanoparticles are more in numbers in the initial sample. These results confirm that the frequency of larger size particles in the river and pond water is higher than the seawater. The UV absorbance of the source water was measured and shown in Fig. 4k. This result shows that the absorbance of the source water follows the order like sea > river > pond, which confirms that the seawater contains smaller size particles than the river and pond water. From TGA, it was observed that solids from the pond showed more weight loss (∼22%) than sea and river water solids (∼15% and ∼10%, respectively) over a temperature range of 40–600 °C (Fig. 4l). In this case (for 40–600 °C), weight loss is directly related to the organic content, and it can be inferred that pond water contains more organic solids or lesser plastic contents.^50^ The organic solids having more plastic content will show high thermal stabilities due to the high thermal resistance of the plastics. Apart from that, the plastics will act as media to carry the hydrophobic organic carbons as suggested by Koelmans *et al.* (2016).^51^ From FTIR analysis, it has been found that the source waters contain plastics, so the thermal stabilities of its solids will get increased. Çepelioǧullar, Ö. & Pütün *et al.* reported that the presence of plastics such as polyethylene terephthalate and polyvinyl chloride had increased the thermal stability of the biomass,^52^ which is similar to the present study. Hence, the solids from sea and river water, which showed increased thermal stabilities may correspond to the presence of more plastic (nano and micro) particles along with other organics.

The real-life field samples from sea, river, and pond were treated by the M-3DPWF system. The physicochemical characterization of the treated water was conducted by zeta size analyzer and UV-vis absorbance measurement. The zeta size analysis of the treated water was performed and is shown in Fig. 4j. Particle size analysis has shown that the average size of the particles in the sea, river, and pond water is 165.1, 372.5, and 510.8 nm, respectively, which confirms that most of the particles are of micro size. The drastic increment in the average size of the particles in all the treated water samples can be attributed to an increased removal of nanosized compared to the micro-sized particles. Furthermore, the UV-vis absorbance (Fig. 4k) of the treated water shows a drastic reduction, which confirms the removal of plastics during the treatment process. Thus, M-3DPWF based treatment system can be successfully employed for field-based real-life water of various sources such as sea, river, and pond.

**Fig. 5:**
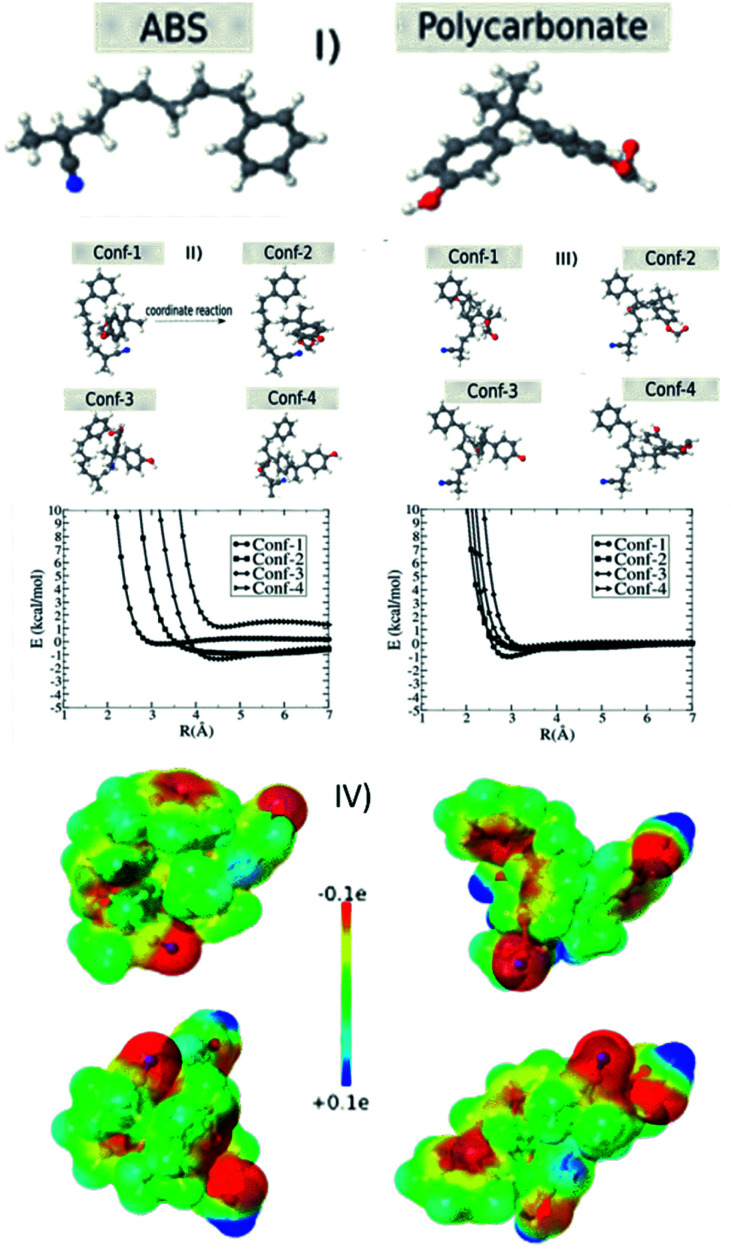
(I) Two prototypal types of the ABS and polycarbonate polymers. The configuration interaction energy between ABS and PC as a function of separation R. For each ABS configuration; (II and III) we considered four different PC configurations. PC is moved along coordinate reaction and configuration interaction energy is calculated as a function of distance separation (*R*) between them. (IV) Detailed electrostatic map for the case of maximum transferred charge.

Generally, the plastics suspended in the polluted water have a surface charge. In order to attract/adhere to the charged particles, we can print the 3D block using oppositely charged materials. In order to have further insights about these aspects at the atomic scale, we carried out semi-empirical quantum simulations using ABS and PC model chains (see Fig. 5I–IV) to understand the removal mechanism concerning the role of surface charge.

As discussed in the materials and methods section, we used PM7 to obtain the optimized geometries, configuration energy (eqn (1)), and charges (electrostatic maps). We tested for other similar parameterizations available in the MOPAC2016, such as PM6 and PM6-DH2, but the results were almost the same. We also tested the solvent effect considering the COSMO method implemented in the MOPAC2016,^53^ assuming that water is modeled as a dielectric medium with a *K* (dielectric constant) equal to 78.4. We verified that the solvent does not significantly affect the total energy values. Fig. 5II–V shows the interaction energy values as a function of separation *R* between ABS and PC molecules. We considered four configurations of the ABS molecule placed at the origin of the axis (Fig. 5II, III and S6†). For each ABS configuration, we consider four different PC configurations. As we can see from Fig. 5II, III and S6† there are not very significant energy variations for the different configurations unless the molecules come very close. In Table 1, we present the heat of formation values for the optimized configurations, considering the ABS as located at the origin reference. In this case, there are energy variations but in the range window of less than 5 kcal mol^−1^. These results show that depending on the configuration, there is a strong ABS/PC interaction. These aspects can be better understood by analyzing the charge transfer and the electrostatic maps of the different configurations. In Table 2, we present the charge profile for the optimized structures from Table 1. The minus sign corresponds to the transferred charge to ABS. Although the specific charge transfer values depend on the configuration (positive and negative), the observed general trend is that the ABS becomes negative. In Fig. 5IV, we present some examples of the electrostatic maps. For these configurations, the transferred charge between ABS and PC was (in me units): −1.36, −4.20, −4.69, and −3.45, respectively. Molar attraction constant is the ability of individual molecules/functional groups to attract another molecule due to intermolecular forces such as dipole–dipole interaction, π–π interaction, and H-bonding. The minus sign means the charge is transferred to ABS. Further, ABS (base material of the M-3DPWF) and PC have higher molar attraction constant (∼1943 and ∼2384 J^1/2^ cm^3/2^ mol^−1^, respectively), which will cause electrostatic attraction among them.^28^

**Table 1:** Heat of formation (kcal mol^−1^)

	conf1	conf2	conf3	conf4
ABS1	−3.00	−2.00	−2.9	−4.72
ABS2	−4.89	−1.52	−1.74	−2.45
ABS3	−0.40	−1.24	−1.40	−3.64
ABS4	−2.65	−4.29	−2.77	−2.78

**Table 2:** Charge transferred

	conf1	conf2	conf3	conf4
ABS1	−1.25	1.30	0.79	−1.36
ABS2	−0.02	−2.98	0.03	−4.20
ABS3	−2.31	−0.35	−4.69	−0.94
ABS4	1.22	−6.24	−3.25	−3.45

The present separation mechanism for the nanoplastics is designed utilizing the electrostatic attraction of the filter material and the charged particles. The size exclusion mechanism depends on the quantity of the surface charge of the suspended particles. We should stress that the DFT calculations used here do not intend to simulate the whole filter process. Even models based on large molecular dynamics simulations cannot incorporate all details of the filter process to be investigated. The DFT simulations were used as a proof of concept and to obtain further insights on specific aspects of the charging behavior due to electrostatic effects caused by the interaction between two prototypes, ABS (base material of the M-3DPWF) and polycarbonate, both present in the filter process. This issue has been hotly debated in the literature sometimes with conflicting results. In order to obtain some insights into the triboelectric effects used on the filter separation process, we recalculated the charge transfer but added one electron (charge = −1*e*) to the system. In this case, the minus sign means the electron excess. We can see from Table 3 that in all cases the tendency is the extra electron to be localized in one of two molecules and of the order of one electron. Thus, we can assume that one of the species remains charged with one electron excess. This observation gives us insights into the possibility of ABS and polycarbonate be used in the filter process through triboelectric separation effects. Thus, the M-3DPWF treatment system can be considered to be a robust process for the removal of nanoplastics by an innovative separation technology.

**Table 3:** Results for charge transfer when the system is charged

		1	2	3	4
conf1	(ABS/polycarbonate)	(−0.99*e*/−0.01me)	(−2.9me/−1.0*e*)	(−10.0me/−0.99*e*)	(−15.4me/0.99*e*)
conf2	(ABS/polycarbonate)	(−17.2me/−0.98*e*)	(−21.3me/−0.98*e*)	(−0.98*e*/−17.2me)	(−11.7me/0.99*e*)
conf3	(ABS/polycarbonate)	(−6.51me/−0.99*e*)	(−6.45me/−0.99*e*)	(−2.6me/−1.0*e*)	(−8.9me/0.99*e*)
conf4	(ABS/polycarbonate)	(−20.4me/−0.98*e*)	(−9.3me/−0.99*e*)	(−10.7me/−0.99*e*)	(−18.0me/0.98*e*)

We carried out fully-atomistic molecular dynamics (MD) simulations with the reactive force field ReaxFF potential,^54^ as implemented in the Large-scale Atomic/Molecular Massively Parallel Simulator (LAMMPS).^55^ We used the parameters set provided by Chenoweth *et al.* for C/H/N/O.^56^ We adopted an NVT ensemble and Nosé–Hoover thermostat.^57^ The equations of motion were numerically integrated using the velocity-Verlet integrator with a time-step of 0.1 fs for a total simulation time of 100 ps.

In order to represent the ABS membrane, we considered that the molecules were pre-optimized at the DFT level. We built the membrane considering the alignment of the molecules along the *x*-direction, in which the membrane is periodic in the *xy*-plane. We considered also cases where the membrane is not infinity (cyclic boundary conditions) along the *y*-direction, see Fig. S7 in the ESI.† The smallest thickness of the ABS membrane is about 4 Å. The separation between the ABS membranes corresponds to 60 Å (Fig. S8 in the ESI†). We performed several simulations for different membranes (types, thickness, and periodic conditions).

Fig. S9† shows representative MD snapshots of the initial configuration at *t* = 0 ps (at left) and the last step corresponding to 100 ps (at right). In Table S2 of the ESI,† we present the relevant parameters for each simulation. We also included the video containing the trajectories for simulations (c), (d), and (f). Simulation of ABS membrane with larger thickness membrane is shown in the Video SV1.† For membranes with the smaller thickness, we observed that ABS molecules are stacked resulting in membrane contraction, as in the MD simulation shown in the Video SV2.† We observed in the last snapshot of all cases the cluster formation with different sizes because of the interaction between the polycarbonates and water molecules. This is in agreement with the experimental data where the particle sizes varying from 1000 to 9000 nm. In all cases, we observed a significant tendency of occurring an attraction between ABS membranes and the polycarbonate plus water clusters. I should be stressed that in all cases the majority of the clusters is attracted to the ABS membrane. This corroborates our interpretation of the results discussed in this manuscript where we analyzed the interaction energy between ABS and polycarbonate. Therefore these results further validate the experimental conclusions that ABS membranes can be a good plastic absorber in an aqueous environment.

## 4 Conclusions

In summary, considering the difficulties associated with the remediation of nanoplastics by conventional filtration processes, a novel 3D printed moving bed water filter has been developed with the opposite surface charged ABS media, selectively trapping the nanoplastics from the water. The M-3DPWF exhibited good compressive strength, yield strength, and toughness due to its ability to easily transfer the load due to its complex topology. The filter effectively works in both static and dynamic conditions. The microscopic and spectroscopic characterization of the initial and treated water indicated a significant removal of the nanoplastics (including polycarbonates). This advanced filtration technique M-3DPWF can be used on a large scale to remove micro and nanoplastics in the source water. The M-3DPWF is very light, can be easily fabricated in large-size dimensions, and can work in different media (sea, river, and pond waters) and in different working conditions (static and dynamical). Furthermore, the scaling-up of M-3DPWF with conventional biological processes (like activated sludge process, moving bed biofilm reactor, sequencing batch reactor, *etc.*) and physicochemical processes (like adsorption, advanced oxidation processes, *etc.*) can be instrumental to treat the industrial wastewater. The results represent a significant low-cost, energy-efficient and feasible advanced system addressing the problem of nanoplastics removal.

## Author contributions

BG, RSA, RS, PK, AM performed experiments, RT, DG performed theoretical work, PMA, PG, DG, AG and CST supervised the project. All authors contributed in analysis and drafting the manuscript.

## Conflicts of interest

There are no conflicts to declare.

## Supplementary Material

RA-011-D1RA03097C-s001

RA-011-D1RA03097C-s002

RA-011-D1RA03097C-s003
