# Supplementary material for: Development of a schwarzite-based moving bed 3D printed water treatment system for nanoplastic remediation
Source: RSC Adv. 2021 Jun 1;11(32):19788–96. doi: 10.1039/d1ra03097c (PMC9033670; doi:10.1039/d1ra03097c)
Supplement: RA-011-D1RA03097C-s001 [file RA-011-D1RA03097C-s001.pdf]

Electronic Supplementary Material (ESI) for RSC Advances.
This journal is © The Royal Society of Chemistry 2021

Supplementary information

Development of *schwarzite*-based moving bed 3D printed water treatment system for nanoplastics remediation

Bramha Gupta [a], Rushikesh S. Ambekar [b], Raphael M. Tromer [c], Partha Sarathi Ghosal [a], Rupal Sinha [a], Abhradeep Majumder [d], Partha Kumbhakar [b], P. M. Ajayan [e], Douglas S. Galvao [c*], , Ashok Kumar Gupta [f*], Chandra Sekhar Tiwary [b*]

[a] School of Water Resources, Indian Institute of Technology Kharagpur, Kharagpur 721302, India

[b] Metallurgical and Materials Engineering, Indian Institute of Technology Kharagpur, Kharagpur 721302, India

[c] Applied Physics Department, State University of Campinas – UNICAMP, 13083-859 - Campinas, SP – Brazil UNICAMP, Brazil

[d] School of Environmental Science and Engineering, Indian Institute of Technology Kharagpur, Kharagpur, 721302, India

[e] Department of Materials Science and Nanoengineering and Smalley-Curl Institute, Rice University, Houston, Texas 77005, United States

[f] Environmental Engineering Division, Department of Civil Engineering, Indian Institute of Technology Kharagpur, Kharagpur 721302, India

*Corresponding author.

E-mail addresses: agupta@civil.iitkgp.ac.in (A.K. Gupta), galvao@ifi.unicamp.br (D.S. Galvao), chandra.tiwary@metal.iitkgp.ac.in (C.S. Tiwary)

Table S1. Comparison of M-3DPWF with other reported polymer and ceramic based composite on the basis of its structural ability.

**Material**	**Compressive strength (MPa)**	**Water absorption (%)**	**Toughness (10^{-6} J/m^3)**	**Young's modulus (MPa)**	**Reference**
Porous ABS filter	3.96	13.42% in 1 day	0.1045	12.22	Present work
Porous photo-polymerizable resin (Spot-E)	0.15				[1]
Poly(ε-caprolactone)/Hydroxyapetite/calcium sulphate	2.64 ± 0.18	40% in 7 days	0.26 ± 0.09	63.39 ± 4.92	[2]
Dolamite/geopolymer foam	6.92 ± 0.16	45.4% in 7 day			[3]
Porous PLA	5.10				[4]
Coal gangue microsphere/ geopolymer composite foam	5.70 ± 0.88				[5]

S1. Sample preparation for FTIR and SEM analysis

The FTIR analysis of the source, raw, and treated water was carried out using FTIR spectroscopy (Nicolet-6700, Thermo Fisher, USA), having wavenumbers ranging from 250 to 4250 cm^{-1}. The sample preparation for FTIR analysis involves the drop casting of liquid sample on the salt plate (KBr), spreading the liquid sample on the salt plate, and then drying it. Then the FTIR analysis of the drop casted salt plate was carried out. The surface morphology was assessed FE-SEM analysis (ZEISS-MERLIN, GEMINI-2, Germany). The sample preparation for FE-SEM analysis involves the drop-casting of raw and treated water sample on the aluminium foil (1x1 cm) sheet, oven drying of the

casted sheet, affixing the casted sheet on carbon tape, and coated with gold to eliminate the charge. The prepared sample was then imaged for FE-SEM analysis."

S2. FTIR analysis

The FTIR spectra (Fig. S1a, d, and e) of the initial and treated water show the presence of CH_2 (near 520 cm^{-1}), C—H, and C—O—C (1252 and 1385 cm^{-1}), C=O, and C=C (1636, 1786, and 2080 cm^{-1}), and N—H, O—H, C—H (3100-3700 cm^{-1}), and CH_2 and CH_3 (2918 and 2928 cm^{-1}) bonds.[6–11] These functional groups belong to the chemical bonds present in the polycarbonate plastics solution. Furthermore, the FTIR spectra of the source water (two samples for each, i.e., sea, river, and pond) (Fig. S1b, c, and f) have shown the presence of the Si—C (near 600 cm^{-1}), CH_2, and C—H (680 cm^{-1}), C—C, C—H, and C—O (880 cm^{-1}), CF_2 and CH_2 (1100-1200 cm^{-1}), C—O—C (1252 cm^{-1}), ester group and C—H (1390 and 1405 cm^{-1}), C—C, C=C, and C=O (1637, 1646, 1800, and 1814 cm^{-1}), N—H (1637 and 1646 cm^{-1}), CH_2 (2920 cm^{-1}), and N—H, O—H, and C—H (3100-3700 cm^{-1}).[6–13] The presence of these bonds might be attributed to Polyethylene (C—H bond at 680, 1390, and 1405 cm^{-1}), Polypropylene (C—H bond at 880, 1405, and 3100-3700 cm^{-1}; C—C bond at 880 cm^{-1}; and CH_2 bond at 1100-1200 cm^{-1}), Polyvinyl Chloride (C=O bond at 1637, 1646, and 1814 cm^{-1}; C—H bond at 1390, 1405 cm^{-1}, and 3100-3700 cm^{-1}), Nylon-6 (N—H bond at 1637, 1646, and 3100-3700 cm^{-1}; C=O bond at 1637, 1646, and 1814 cm^{-1}), Polystyrene (C—H bond at 680, 880, 1405, and 3100-3700 cm^{-1}; C—C bond at 1637 and 1646 cm^{-1}), and Polycarbonate (CH_2 bond at 680 cm^{-1} and 2920 cm^{-1}; C—O—C bond at 1252 cm^{-1}; C—H bond at 1390 and 3100-3700 cm^{-1}; C=O and C=C bond at 1637, 1800, and 1814 cm^{-1}; N—H bond at 1637, 1646, and 3100-3700 cm^{-1}; and O—H bond at 3100-3700 cm^{-1}).[11,14–16]. The FTIR spectra for two samples of sea,

river, and pond water are not alike, which may be due to the heterogeneous distribution of the plastic particles in these two samples.

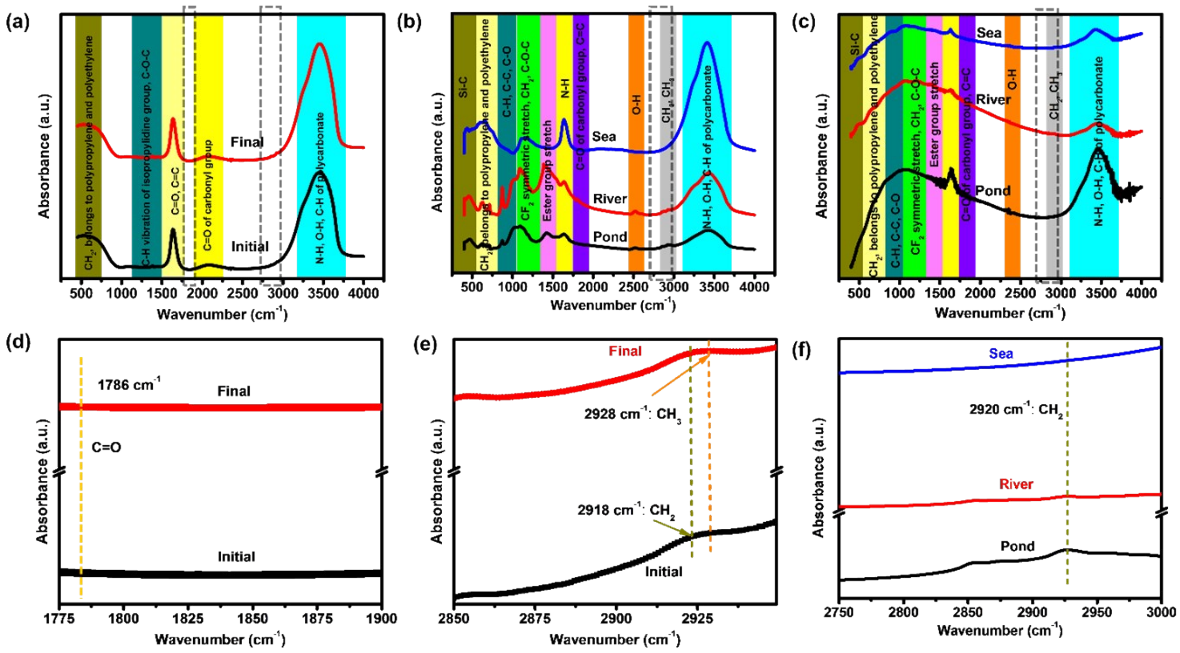

Fig. S1. (a) Fourier transform infrared spectra show the presence of functional groups in polycarbonate contaminated water at the initial and final stages. **(b and c)** Fourier transform infrared spectra show the presence of functional groups in the source water (For two samples taken from two locations of the sampling bottle). **(d, e, and f)** Enlarged view of FTIR spectra (for a and b & c, respectively) in the ranges of 1775-1900, 2850-3000, and 2750-3000 cm^{-1}, respectively.

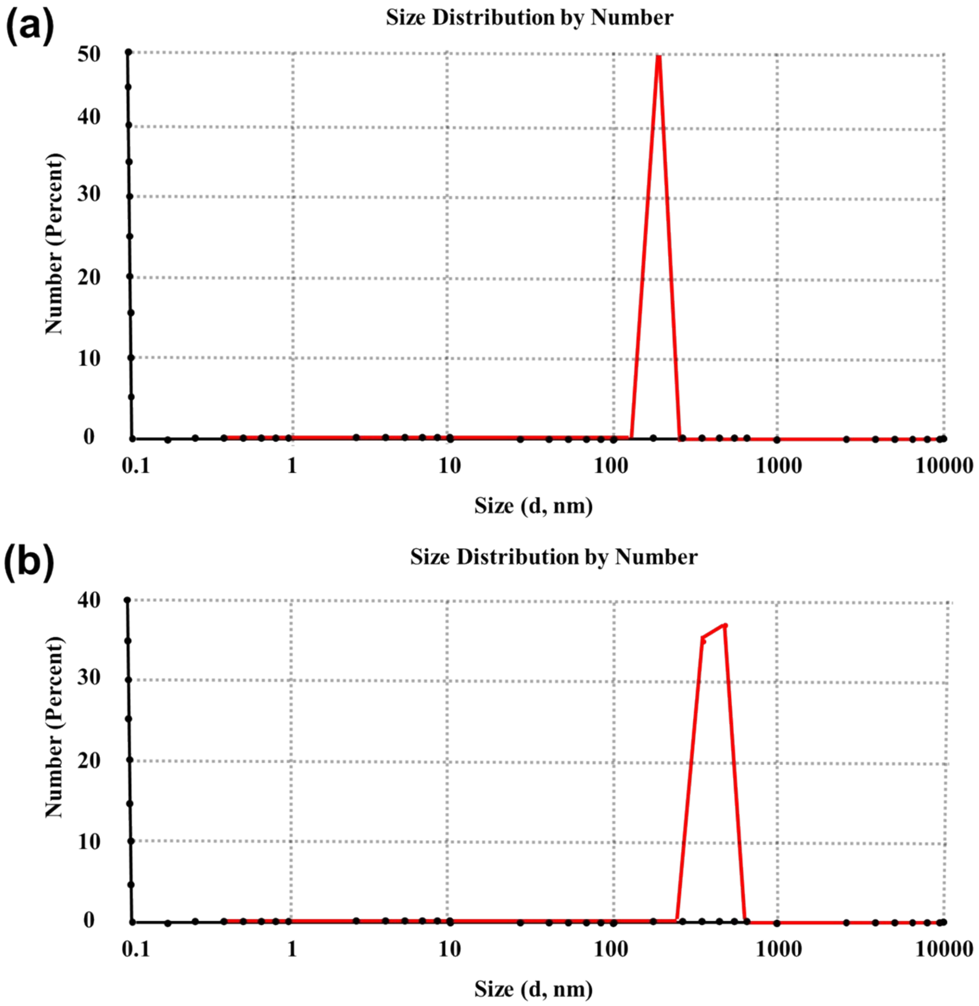

Fig. S2. Size distribution by number of the particles in the **(a)** raw and **(b)** treated water.

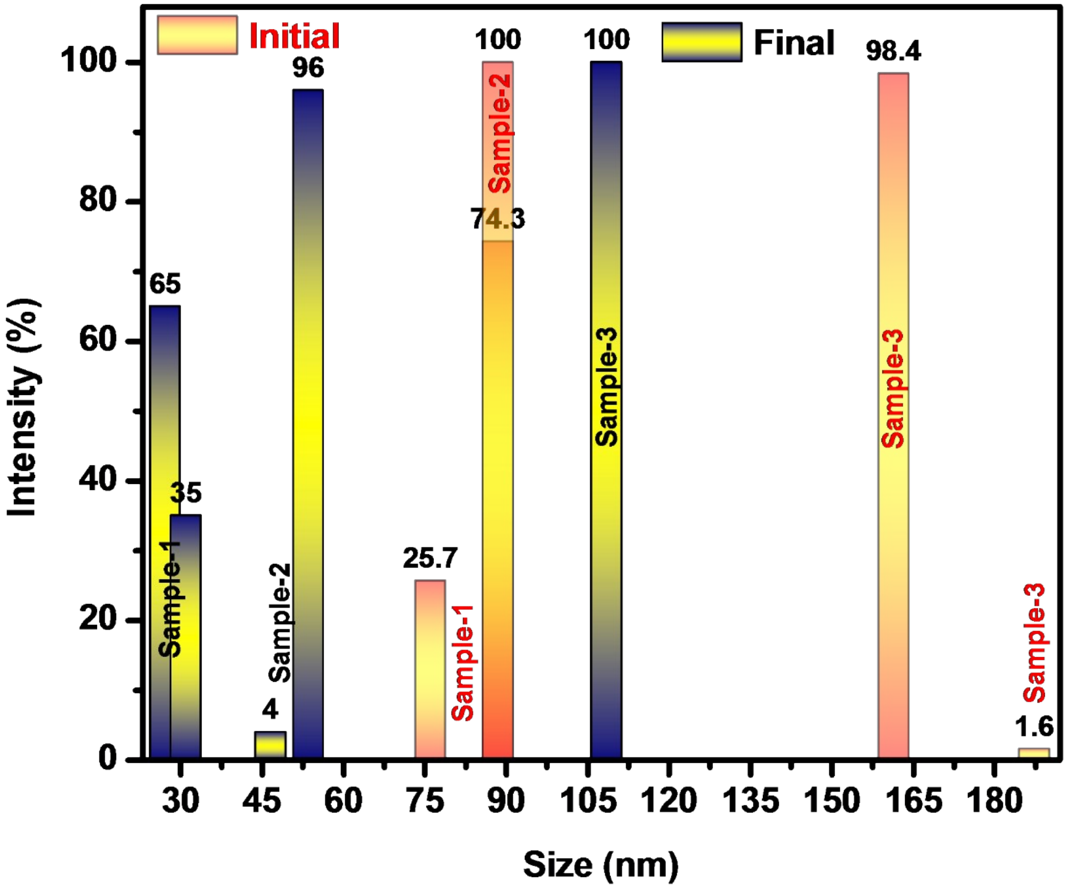

Fig. S3. Zeta size analysis of three samples of raw and treated water collected from various depth from the surface of the sample.

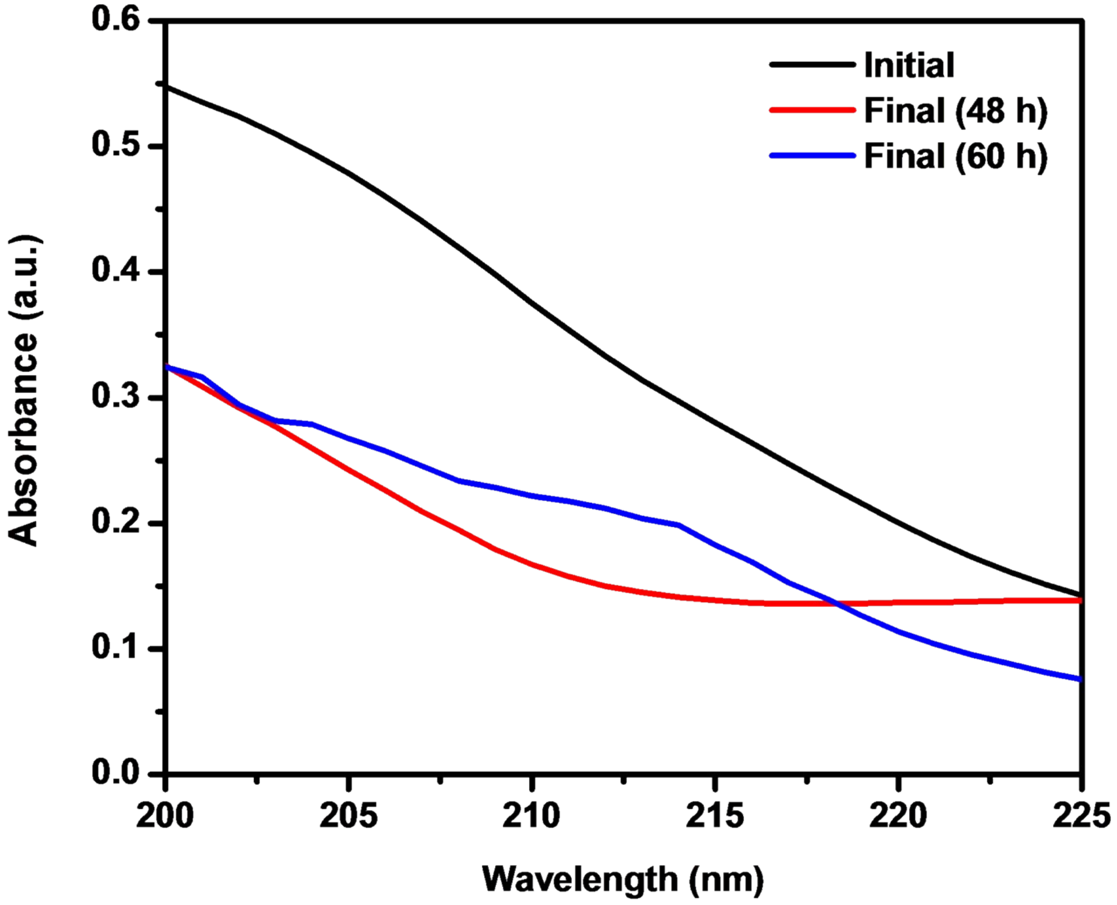

Fig. S4. UV absorbance of the initial and treated water samples (at 48 h and 60 h).

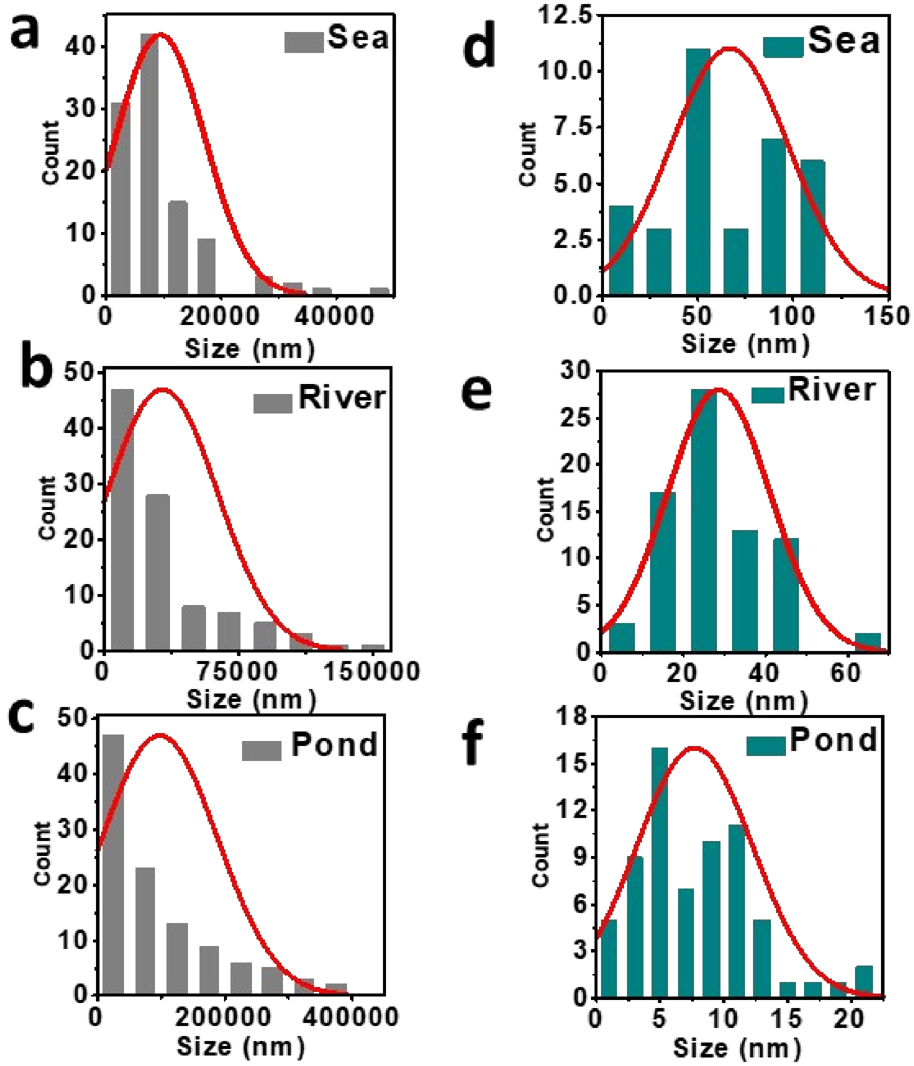

Fig. S5. (a, b, and c) Histogram of the FESEM of sea, river, and pond water calculated from. (d, e, and f) Histogram of the TEM images of sea, river, and pond water.

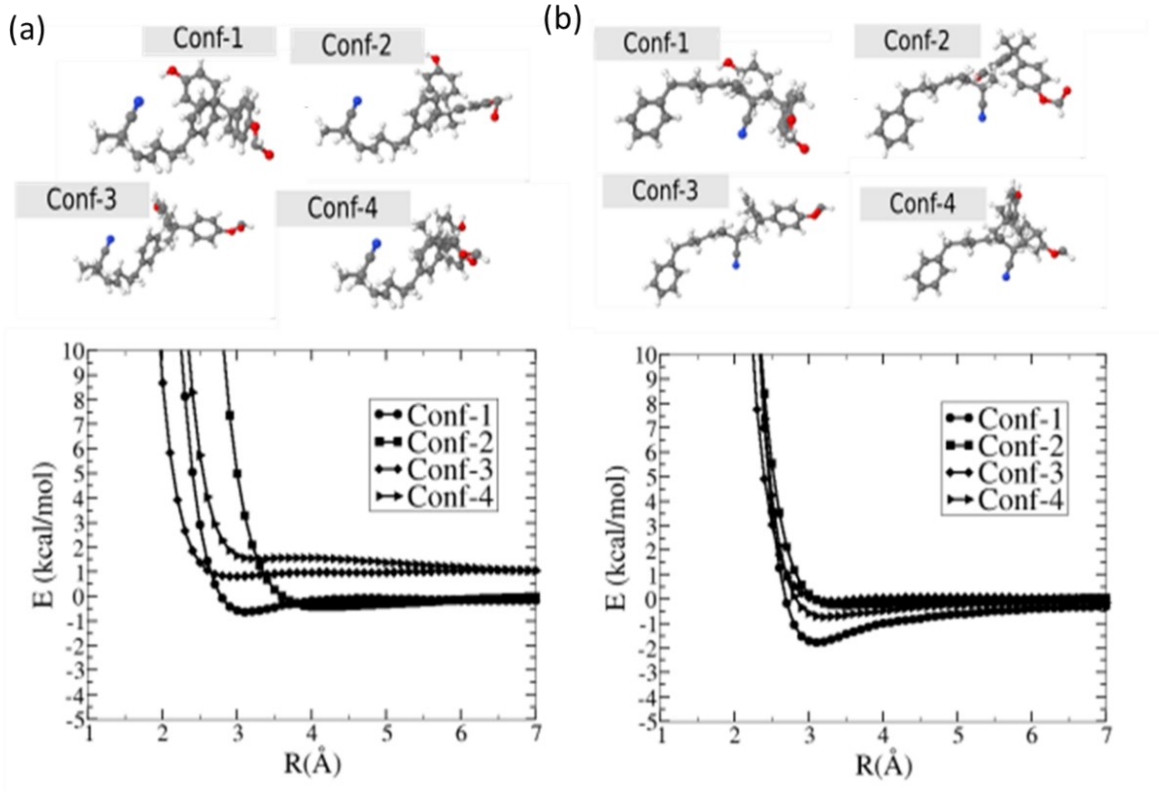

Fig. S6. (a-b) we considered four different PC configurations. PC is moved along coordinate reaction and configuration interaction energy is calculated as a function of distance separation.

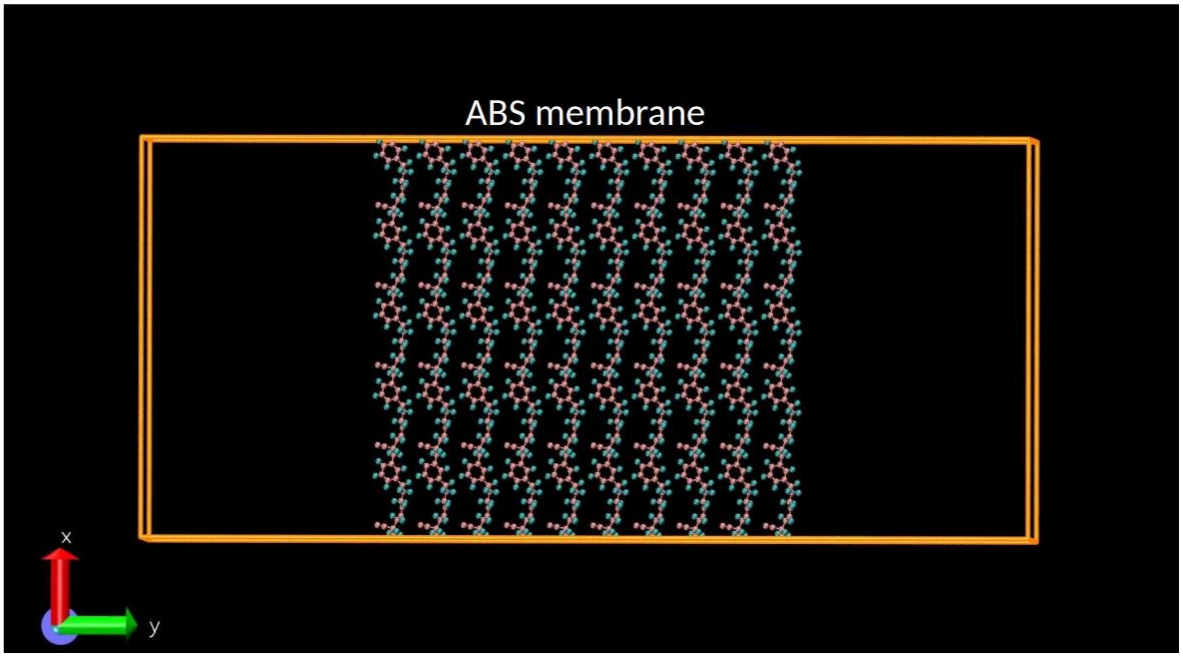

Fig. S7. An ABS membrane composed of aligned molecules along the x-direction. We considered the ABS membrane periodic only along the x-direction and, also in the xy-plane.

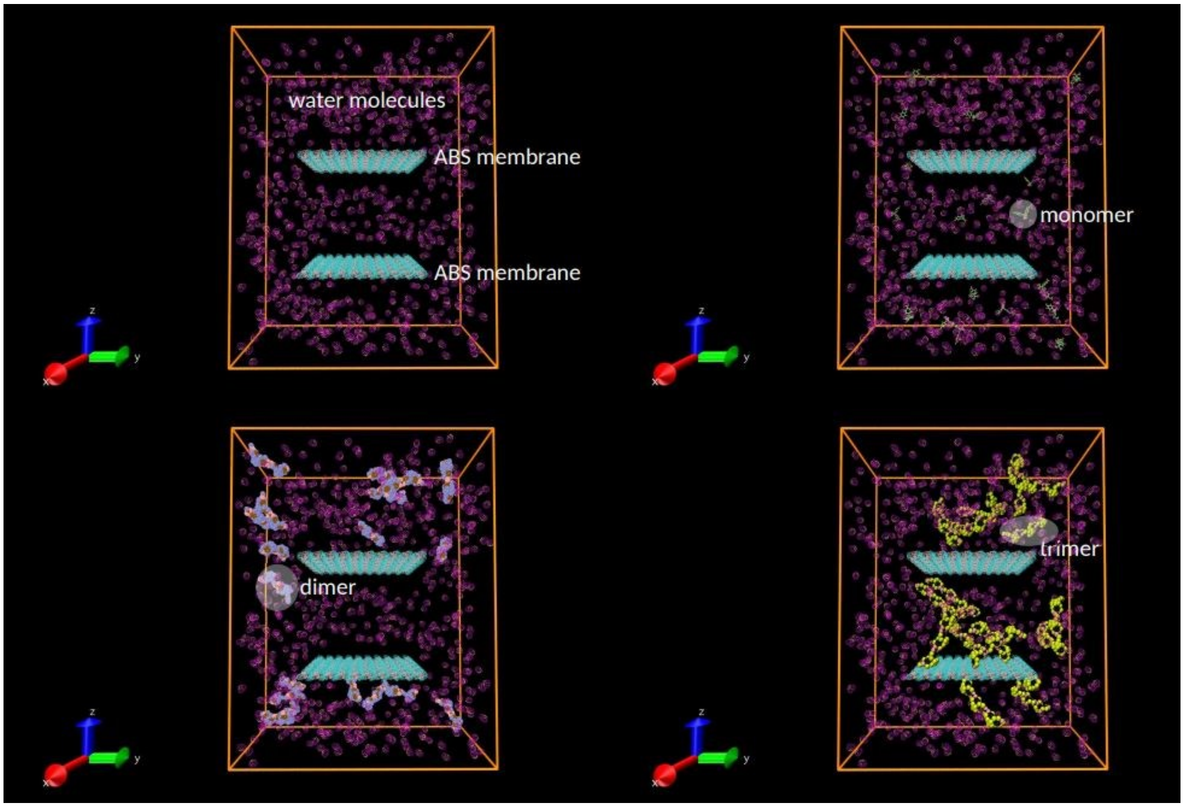

Fig. S8. Inside the simulation box, we have ABS membranes, water molecules, monomers, dimers and, trimers of polycarbonate chains.

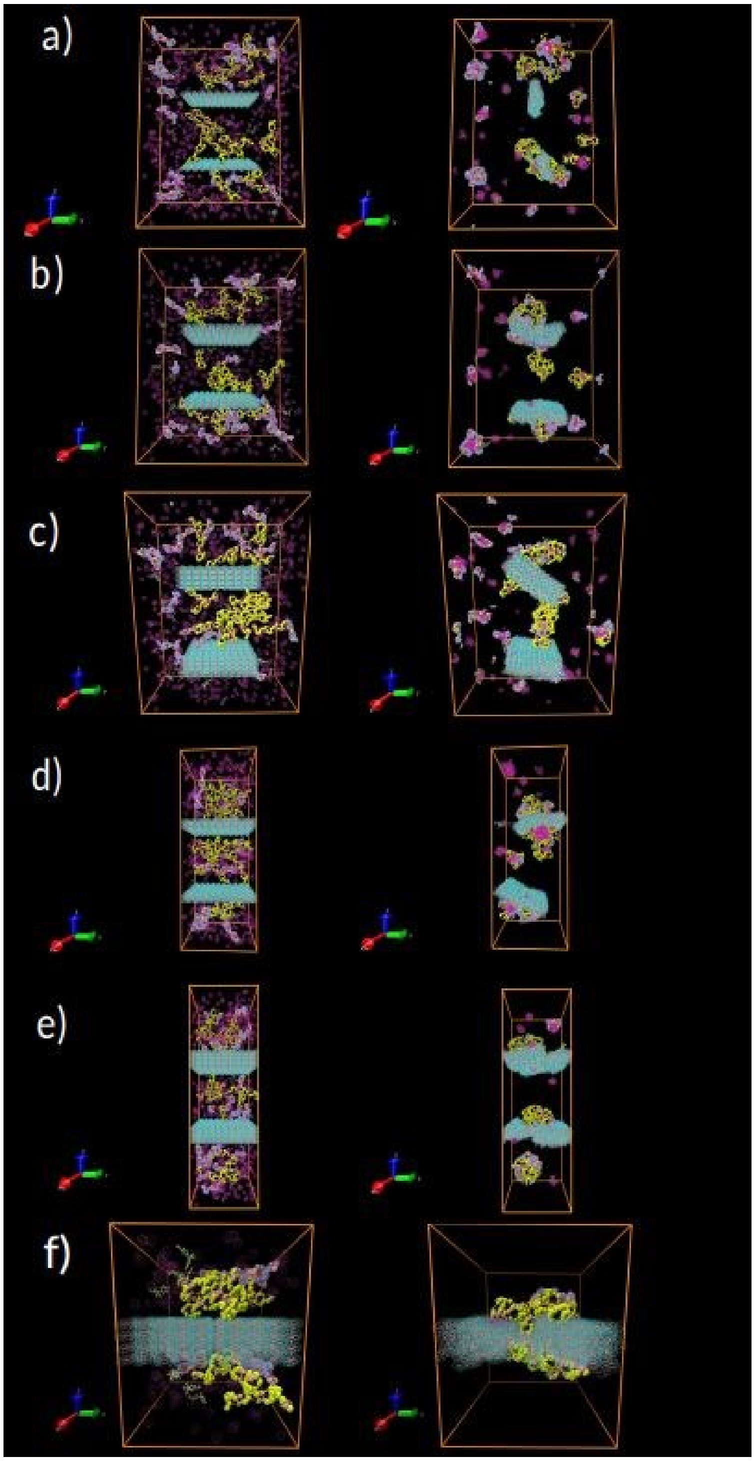

Fig. S9. Representative MD snapshots from NVT simulations considering different ABS membrane types. From a) to c) the ABS membranes are periodic only along the x-direction and xy for other cases.

Table S2. Parameters used in the MD simulations shown in Figure S9.

Label	Num. of atoms	L_x(Å)	L_y(Å)	L_z(Å)	Thickness (Å)	Membrane Periodicity
a	8280	63.05	140	180	4	X
b	12840	63.05	140	180	8	X
c	19440	63.05	140	180	16	X
d	19440	63.05	68.1	220	8	x-y
e	12840	63.05	68.1	180	16	x-y
f	9540	63.05	68.1	80	16	x-y

Supplementary Videos

SV1: NVT molecular dynamics considering ABS membrane with larger thickness.

SV2: NVT molecular dynamics considering ABS membrane with smaller thickness.

References

1 X. Mu, T. Bertron, C. Dunn, H. Qiao, J. Wu, Z. Zhao, C. Saldana and H. J. Qi, *Mater. Horizons*, 2017, **4**, 442–449.

2 Z. Zhou, E. Cunningham, A. Lennon, H. McCarthy, F. Buchanan and N. Dunne, *Mater. Sci. Eng. C*, 2018, **93**, 975–986.

3 S. Yan, F. Zhang, S. Wang, P. He, D. Jia and J. Yang, *Cem. Concr. Compos.*, 2019, **104**, 103376.

4 S. M. Sajadi, P. S. Owuor, S. Schara, C. F. Woellner, V. Rodrigues, R. Vajtai, J. Lou, D. S. Galvão, C. S. Tiwary and P. M. Ajayan, *Adv. Mater.*, 2018, **30**, 1704820.

5 S. Yan, F. Zhang, J. Liu, B. Ren, P. He, D. Jia and J. Yang, *J. Clean. Prod.*, 2019, **227**, 483–494.

6 A. Cincinelli, C. Scopetani, D. Chelazzi, E. Lombardini, T. Martellini, A. Katsoyiannis, M. C. Fossi and S. Corsolini, *Chemosphere*, 2017, **175**, 391–400.

7 L. Tremblay, G. Alaoui and M. N. Léger, *Environ. Sci. & Technol.*, 2011, **45**, 9671–9679.

8 A. B. Castillo, I. Al-Maslamani and J. P. Obbard, *Mar. Pollut. Bull.*, 2016, **111**, 260–267.

9 B. Gupta, A. K. Gupta, P. S. Ghosal and C. S. Tiwary, *Environ. Res.*, 2020, 109154.

10 A. Käppler, F. Windrich, M. G. J. Löder, M. Malanin, D. Fischer, M. Labrenz, K. J. Eichhorn and B. Voit, *Anal. Bioanal. Chem.*, 2015, **407**, 6791–6801.

11 M. G. J. Löder, M. Kuczera, S. Mintenig, C. Lorenz and G. Gerdts, *Environ. Chem.*, 2015, **12**, 563–581.

12 J. Feng, J. Hao, J. Du and R. Yang, *Polym. Degrad. Stab.*, , DOI:10.1016/j.polymdegradstab.2012.01.011.

13 C. Ioakeimidis, K. N. Fotopoulou, H. K. Karapanagioti, M. Geraga, C. Zeri, E. Papathanassiou, F. Galgani and G. Papatheodorou, *Sci. Rep.*, , DOI:10.1038/srep23501.

14 A. S. Tagg, M. Sapp, J. P. Harrison and J. J. Ojeda, *Anal. Chem.*, 2015, **87**, 6032–6040.

15 E. Apaydin-Varol, S. Polat and A. E. Putun, *Therm. Sci.*, 2014, **18**, 833–842.

16 K. L. Ng and J. P. Obbard, *Mar. Pollut. Bull.*, 2006, **52**, 761–767.
