# Supplementary figures and images for: Development of a schwarzite-based moving bed 3D printed water treatment system for nanoplastic remediation
Source: RSC Adv. 2021 Jun 1;11(32):19788–96. doi: 10.1039/d1ra03097c (PMC9033670; doi:10.1039/d1ra03097c)

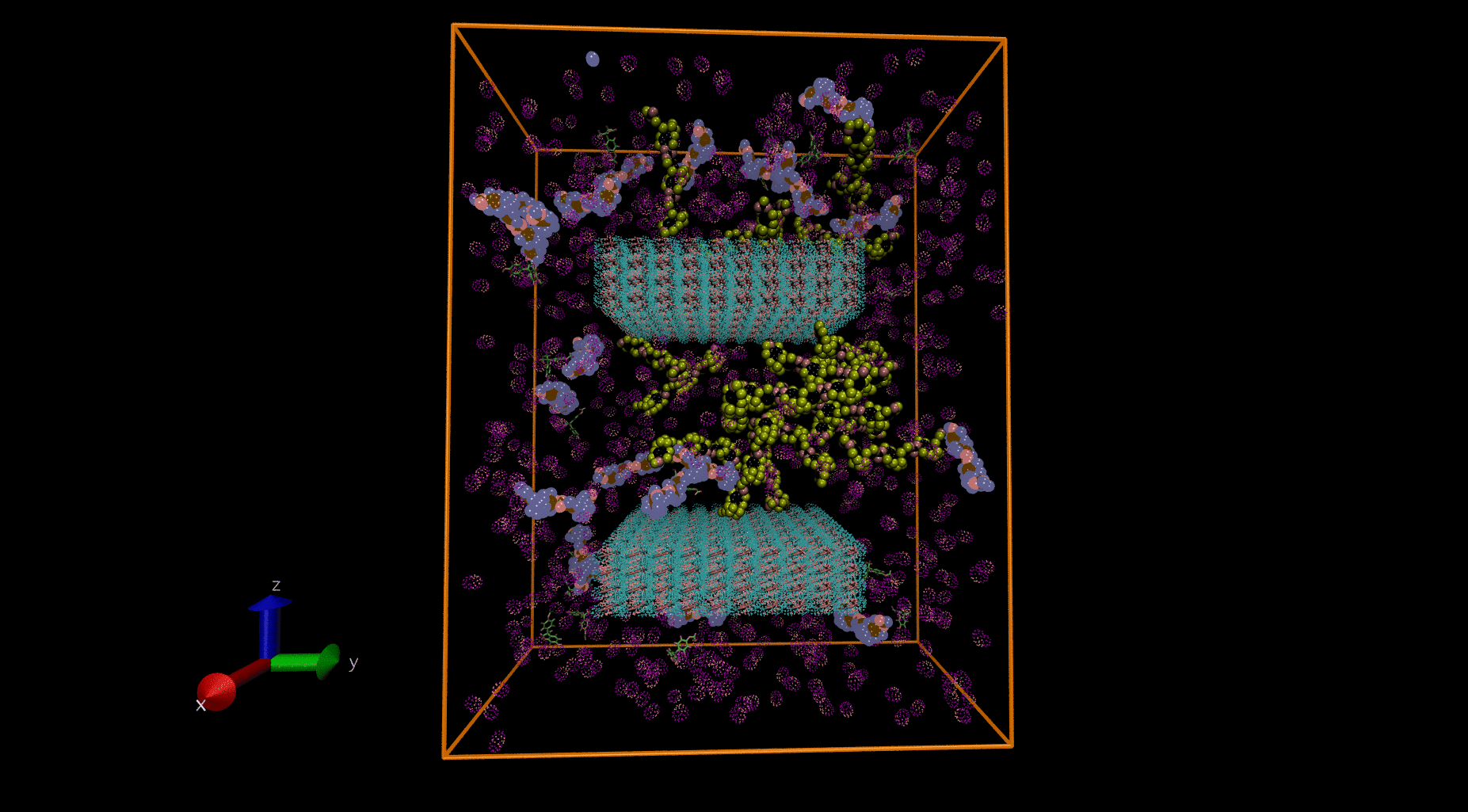

Supplement: RA-011-D1RA03097C-s002 [file RA-011-D1RA03097C-s002.gif]

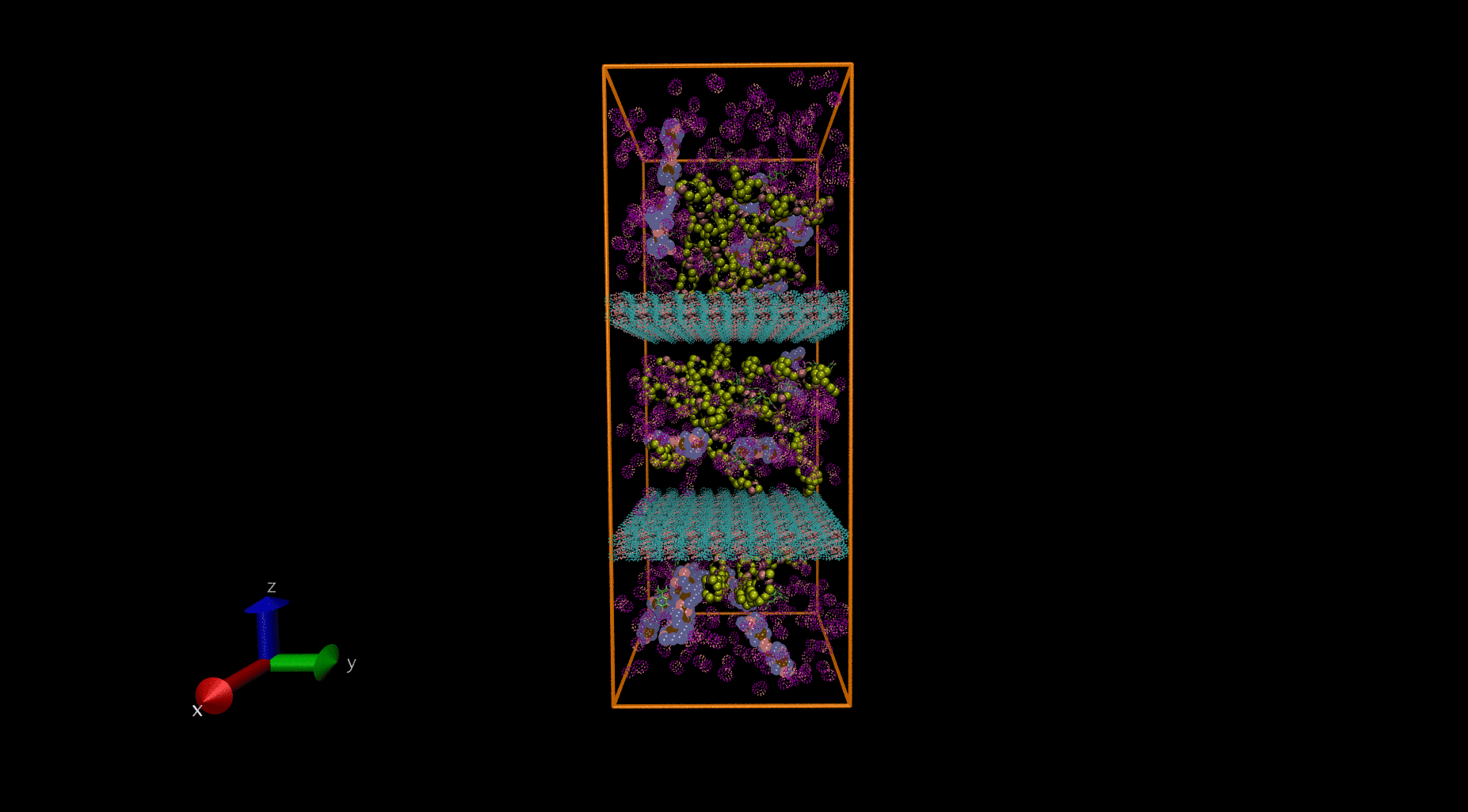

Supplement: RA-011-D1RA03097C-s003 [file RA-011-D1RA03097C-s003.gif]
